# Proximal junctional kyphosis is a compensation for post-operative negative C2-FH in ASD patients: a cross-sectional study

**DOI:** 10.1186/s13018-022-03336-6

**Published:** 2022-10-07

**Authors:** Xin Zhang, Shibin Shu, Zezhang Zhu, Qi Gu, Zhen Liu, Yong Qiu, Hongda Bao

**Affiliations:** 1 Division of Spine Surgery, Department of Orthopedic Surgery, Nanjing Drum Tower Hospital, The Affiliated Hospital of Nanjing University Medical School, Nanjing, China; 2 Division of Spine Surgery, Department of Orthopedic Surgery, Nanjing Drum Tower Hospital, Clinical College of Nanjing Medical University, Nanjing, China

**Keywords:** Proximal junctional kyphosis, Adults spinal deformity, Spinal deformity, C2-FH, Sagittal global balance

## Abstract

**Background:**

Recent studies have found that C2-FH is close to 0 cm in both standing and sitting position for asymptomatic adults. We hypothesize that the thoracic spine may compensate with PJK when the immediate post-operative C2-FH was not ideally restored in adult spinal deformity (ASD).

**Methods:**

The inclusion criteria were as follows: ASD patients over 45 years old; Cobb angle > 30°; with posterior spinal correction surgery; at least 2 years follow-up. C2-FH was defined as the distance between the femoral heads to the C2 vertical line. All participants were divided into two groups according to the occurrence of PJK at the last follow-up: PJK group and non-PJK group.

**Results:**

68 ASD patients, with a minimum follow-up of 2.5 years, were included. PJK was found in 24 patients (35.3%) while the rest 44 patients remained no sagittal malalignment. Immediately post-operative C2-FH showed significant difference between PJK group and non-PJK group (*p* = 0.015). However, at the last follow-up, C2-FH showed no significant difference between PJK and non-PJK group and the mean value of C2-FH in both groups was approximately − 1 cm, indicating that ASD patients could develop various compensatory mechanisms to maintain sagittal global balance. The AUC was 0.84 (95%CI 0.68–0.97), indicating the well effectiveness of ROC curve and cut-off value in predicting occurrence of PJK in ASD patients. Based on the ROC curve, the optimal cut-off value of C2-FH as indicators for occurrence of PJK was − 42.3 mm.

**Conclusion:**

Immediate postoperative negative global malalignment (C2-FH < − 42.3 mm) may predict proximal junctional kyphosis in ASD patients. The normal value of C2-FH, − 1 cm, may be the target of global sagittal compensation, and PJK is a compensatory mechanism.

*Trial registration*: 2021-LCYJ-DBZ-05, 2021.07, Retrospective study.

## Introduction

Optimal surgical correction for adult spinal deformity (ASD) is essential in ameliorating sagittal decompensation and improving clinical outcomes [1, 2]. However, unexpected complications such as proximal junctional kyphosis (PJK) are not uncommon after surgical restoration.

PJK was originally described as a radiographic manifestation, the incidence of which could be as high as 62%. Several studies have tried to evaluate the incidence, risk factors, and pathogenic mechanisms of PJK [3–6]. Kim et al. reported risk factors for the development of PJK, including fusions to the sacrum, age, and overcorrection of lumbar lordosis [7]. In addition, inadequate restoration of global sagittal alignment was also reported as a potential risk factor by Yagi et al. [6, 8]. However, the risk factor of PJK is still unclear.

Studies have shown that ASD patients with upper instrumented vertebrae (UIV) located at lower thoracic vertebrae (T8-L1) have a higher incidence of PJK than patients with UIV located at upper thoracic vertebrae (T1–T7), indicating that the unfused segments of the thoracic vertebrae had a certain compensatory capacity [9]. In a sense, the development and progression of PJK may be a compensatory mechanical in the case of residual post-operative global malalignment.

**Fig. 1:**
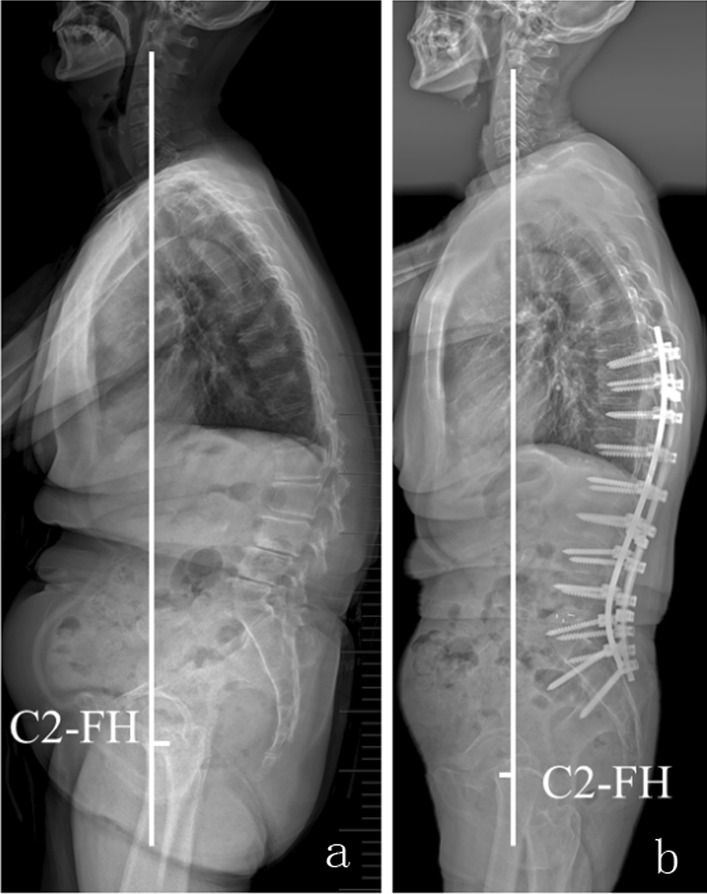
C2-FH is defined as the distance between the femoral heads to the C2 vertical line. Preoperative C2-FH (**a**) and postoperative C2-FH (**b**)

Previous studies took a certain range of T1 pelvic angle (TPA < 14°) and sagittal vertical axis (SVA < 4 cm) as an important reference for sagittal restoration in the surgical planning for ASD [10, 11]. However, the SVA and TPA are variable in the population [12], and the ideal range of these parameters is too large to be established as the target of correction. Recently, the global sagittal alignment parameter C2-FH (defined as the distance between the femoral heads to the C2 vertical line, Fig. 1) was found to be constant in both sitting and standing positions for asymptomatic individuals. Thus, the consistent C2-FH may be used as a surgical target for the sagittal restoration of ASD. We hypothesize that the thoracic spine may compensate with PJK when the immediate post-operative C2-FH was not ideally restored, and that negative C2-FH could predict the development of PJK.

## Materials and methods

### Subjects

68 ASD patients who underwent correction surgery in our spine center from February 1, 2017, to February 1, 2018, were analyzed retrospectively. The inclusion criteria were as follows: ASD patients over 45 years old; Cobb angle > 30°; with posterior spinal correction surgery; at least 2 years follow-up. Patients with history of spinal surgeries, tumors or infection were excluded. In addition, patients with incomplete radiological data were also excluded. This study was approved by the Ethics Committee of our hospital. All patients were informed about the risks, purposes and methods involved in the study protocol and signed informed consent.

### Data collection

Demographic data including age, gender and follow-up time were collected. Full-length standing lateral spine radiographs (36″ cassette) at preoperative, immediate postoperative and at follow-up were analyzed using Surgimap (Nemaris Inc., New York, NY, USA). All radiographic measures were performed by a senior spinal surgeon and a radiologist with experience and included the following spino-pelvic parameters: Pelvic Incidence (PI), Pelvic Tilt (PT), Sacral Slope (SS), Lumbar Lordosis between L1 and S1 (LL), Thoracic kyphosis between T4 and T12 (TK), Cervical Lordosis between C2 and C7 (CL), T1-Pelvic-Angle (TPA), Sagittal Vertical Axis (SVA) and the distance between the femoral heads center to C2 vertical line (C2-FH). The Hounsfield units (HU) measured on computed tomography (CT) can be used to estimate the bone mineral density of vertebral bodies [13–16]. The HU value of L1 vertebral body was used in this study. BMI and menopausal status of patients were also statistically analyzed.

In addition to the abovementioned sagittal parameters, the proximal junctional angle (PJA) was also measured, with the definition of the Cobb angle between the lower endplate of the upper most instrumented vertebra (UIV) and the upper endplate of 2 vertebrae above the UIV. All patients were divided into two groups according to the occurrence of PJK: PJK group and non-PJK group.

### Statistics

Data were statistically analyzed and processed using IBM SPSS Statistics version 23.0. After describing the cohort in terms of radiographic alignment and baseline demographic, including classic spinopelvic parameters, PJA, C2-FH, and so on, an immediate postoperative and follow up of PJK group and non-PJK group analysis were conducted using paired *t* test. Independent *t* test was used to analyze the differences between PJK group and non-PJK group at immediate postoperative and follow-up. *χ*^2^ test was used to compare the differences of count data (univariate analysis). Receiver operating characteristics (ROC) curve was performed to determine the optimal cut-off value of PJK as indicators for occurrence of PJK. Mean values were reported as mean (SD). The significance level of all statistical analysis was set as *p* < 0.05.

## Results

68 patients, 6 males and 62 females, with a mean 3.81 years follow-up, were recruited in this study. PJK was found in 24 patients (35.3%) while the rest 44 patients remained no sagittal malalignment during follow-up. The mean age of non-PJK group patients was 57.27 ± 6.51 years (range 46–65 years), while the mean age of PJK group patients was 62.25 ± 5.62 years (range 55–65 years). BMI, HU value and menopausal status were not significantly different between the two groups. Except for SVA (*p* = 0.022), the rest preoperative sagittal parameters showed no significant difference between non-PJK group and PJK group (Table 1). Among all patients, 24 (54.5%) and 8 (33.3%) patients with lower instrumented vertebra (LIV) at L5 in non-PJK group and PJK group, respectively. And 20 (45.5%) and 16 (66.7%) patients with lower instrumented vertebra at pelvis(S1 + S2) in non-PJK group and PJK group respectively, no significant difference were found (*p* = 0.094) (Table 1).

**Table 1:** Comparison of preoperative demographic and radiological data between PJK group and non-PJK group

Parameters	Non-PJK group (*n* = 44)	PJK group (*n* = 24)	*p* value
Age	57.27 ± 6.51	62.25 ± 5.62	0.200
Female (%)	40 (90.9%)	22 (91.7%)	0.835
BMI (kg/m^2^)	25.21 ± 3.58	26.95 ± 3.46	0.057
HU value	150.0 ± 56.02	138.0 ± 59.19	0.411
Menopause	26 (65.0%)	18 (81.8%)	0.176
Follow-up (years)	3.67 ± 1.05	3.92 ± 0.87	0.428
LIV			
L5	24 (54.5%)	8 (33.3%)	–
S1	12 (27.3%)	4 (16.7%)	–
S2	8 (18.2%)	12 (50%)	–
S1 + S2	20 (45.5%)	16 (66.7%)	0.094
Fusion levels	6.91 ± 1.81	8.33 ± 1.37	0.115
C2-FH (mm)	− 6.93 ± 42.15	11.63 ± 35.91	0.116
TK (°)	21.49 ± 12.69	18 ± 13.83	0.652
CL (°)	16.17 ± 11.81	31.8 ± 25.78	0.114
LL (°)	27.4 ± 15.51	14.2 ± 30.14	0.276
SVA (mm)	39.64 ± 41.79	72.08 ± 62.02	0.022***
TPA (°)	21.58 ± 6.94	27.9 ± 5.75	0.384
PI (°)	49.67 ± 9.33	46.55 ± 4.36	0.538
PT (°)	24.23 ± 6.41	31.33 ± 4.77	0.416
SS (°)	25.45 ± 11.15	15.23 ± 5.46	0.215

*C2-FH* the distance between C2 vertical line to the femoral heads, *CL* cervical lordosis, *TK* thoracic kyphosis, *LL* lumbar lordosis, *PT* pelvic tilt, *PI* pelvic incidence, *SS* sacral slope, *SVA* sagittal vertical axis, *TPA* T1-Pelvic angle, *HU* Hounsfield unit, *** p<0.05

The sagittal parameters and surgical data were compared between PJK group and non-PJK group at immediate postoperative (Table 2) and at the last follow-up (Table 4), in order to investigate which parameters may have a predictive effect on PJK. Immediately post-operative C2-FH showed significant difference between PJK group and non-PJK group (*p* = 0.015, Table 2). In addition, immediate postoperative TPA (*p* = 0.006) and PT (*p* = 0.006) also showed significant differences between PJK group and non-PJK group. Logistic regression analysis identified the C2-FH to be the independent factors predicting PJK after surgery (*p* = 0.001, Table 3).

**Table 2:** Comparison of immediate postoperative data between PJK group and non-PJK group

Parameters	Non-PJK group (*n* = 44)	PJK group (*n* = 24)	*p* value
C2-FH (mm)	− 36.64 ± 24.03	− 72.88 ± 29.38	0.015***
TK (°)	24.77 ± 11.09	18.35 ± 7.49	0.226
CL (°)	18.55 ± 13.04	13.55 ± 10.34	0.432
L L(°)	42.62 ± 11.44	32.63 ± 12.58	0.117
SVA (mm)	0.76 ± 16.48	− 10.95 ± 29.01	0.300
TPA (°)	13.45 ± 4.88	20.77 ± 3.77	0.006***
PI (°)	50.07 ± 8.15	48.22 ± 10.45	0.690
PT (°)	19.91 ± 5.18	28.3 ± 4.95	0.006***
SS (°)	30.16 ± 9.6	19.92 ± 12.25	0.075
PJA (°)	3.1 ± 7.96	5.62 ± 3.49	0.500

*C2-FH* the distance between C2 vertical line to the femoral heads, *CL* cervical lordosis, *TK* thoracic kyphosis, *LL* lumbar lordosis, *PT* pelvic tilt, *PI* pelvic incidence, *SS* sacral slope, *SVA* sagittal vertical axis, *TPA* T1-Pelvic angle, *PJA* proximal junctional angle, *** p<0.05

**Table 3:** Logistic regression analysis of risk factors for proximal junctional kyphosis after surgery

Risk factors	B	SE	Wald	*p* value	Odds ratio (95% CI)
TPA	0.230	0.134	2.933	0.087	1.258 (0.967–1.636)
PT	0.182	0.105	2.993	0.084	1.200 (0.976–1.474)
C2-FH	− 0.110	0.032	11.748	0.001***	0.896 (0.842–0.954)
Constant	− 13.883	3.939	12.423	0.000***	0.00

*C2-FH* the distance between C2 vertical line to the femoral heads, *SVA* sagittal vertical axis, *TPA* T1-Pelvic angle, *** p<0.05

During follow-up, with the occurrence of PJK, LL (*p* = 0.043), TPA (*p* < 0.000), PT (*p* < 0.000) and SS (*p* = 0.002) indicated significant differences between PJK group and non-PJK group. However, C2-FH showed no significant difference between PJK group and non-PJK group and the mean value of C2-FH in both groups was approximately − 1 cm (Table 4). The above results suggested that a C2-FH of − 1 cm may be the target of compensation for ASD patients, but with different compensatory mechanisms.

**Table 4:** Comparison of follow-up data between PJK group and non-PJK group

Parameters	Non-PJK group (*n* = 44)	PJK group (*n* = 24)	*p* value
C2-FH (mm)	− 17.96 ± 12.24	− 11.78 ± 23.92	0.486
TK (°)	26.38 ± 14.79	28.42 ± 10.87	0.772
CL (°)	20.03 ± 11.71	32.83 ± 18.45	0.098
LL (°)	38.84 ± 13.25	23.55 ± 14.28	0.043***
SVA (mm)	18.21 ± 23.55	45.3 ± 58.89	0.192
TPA (°)	16.81 ± 5.74	33.13 ± 6.03	< 0.000***
PI (°)	49.84 ± 7.71	50.14 ± 9.64	0.945
PT (°)	22.02 ± 5	37.13 ± 5.21	< 0.000***
SS (°)	27.82 ± 8.4	13 ± 7.23	0.002***
PJA (°)	9.92 ± 8.91	20.47 ± 7.41	0.06

*C2-FH* the distance between C2 vertical line to the femoral heads, *CL* cervical lordosis, *TK* thoracic kyphosis, *LL* lumbar lordosis, *PT* pelvic tilt, *PI* pelvic incidence, *SS* sacral slope, *SVA* sagittal vertical axis, *TPA* T1-Pelvic angle, *PJA* proximal junctional angle, *** p<0.05

The sagittal parameters were also compared between immediate postoperative and follow-up in both PJK group (Table 5) and non-PJK group (Table 6), in order to investigate the prognosis of C2-FH from immediate postoperative to follow-up in ASD patients with or without PJK and the compensatory mechanism of spine and pelvis after the occurrence of PJK. All parameters except PI and SVA showed significant differences between immediate postoperative and follow-up in PJK group (Table 5). The most noteworthy finding is that C2-FH was close to the ideal value (− 1 cm) at follow up. In the non-PJK group, only C2-FH showed significant difference between immediate postoperative and follow-up (*p* = 0.022, Table 6), indicating that a small change of C2-FH could be compensated without PJK.

**Table 5:** Comparison of data between immediate postoperative and follow-up in the PJK group

Parameters	Immediate postoperative	Follow up	*p* value
C2-FH (mm)	− 72.88 ± 29.38	− 11.78 ± 23.92	0.004***
TK (°)	18.35 ± 7.49	28.42 ± 10.87	0.033***
CL (°)	13.55 ± 10.34	31.67 ± 16.54	0.046***
LL (°)	32.63 ± 12.58	23.55 ± 14.28	0.008***
SVA (mm)	− 10.95 ± 29.01	45.3 ± 58.89	0.057
TPA (°)	20.77 ± 3.77	33.13 ± 6.03	0.002***
PI (°)	48.22 ± 10.45	50.14 ± 9.64	0.222
PT (°)	28.3 ± 4.95	37.13 ± 5.21	0.008***
SS (°)	19.92 ± 12.25	13 ± 7.23	0.040***
PJA (°)	5.62 ± 3.49	20.47 ± 7.41	0.001***

*C2-FH* the distance between C2 vertical line to the femoral heads, *CL* cervical lordosis, *TK* thoracic kyphosis, *LL* lumbar lordosis, *PT* pelvic tilt, *PI* pelvic incidence, *SS* sacral slope, *SVA* sagittal vertical axis, *TPA* T1-Pelvic angle, *PJA* proximal junctional angle, *** p<0.05

**Table 6:** Comparison of data between immediate postoperative and follow-up in the no PJK group

Parameters	Immediate postoperative	Follow up	*p* value
C2-FH (mm)	− 36.64 ± 24.03	− 17.96 ± 12.24	0.022***
TK (°)	24.77 ± 11.09	26.38 ± 14.79	0.433
CL (°)	18.55 ± 13.04	20.03 ± 11.71	0.678
LL (°)	42.62 ± 11.44	38.84 ± 13.25	0.127
SVA (mm)	0.76 ± 16.48	18.21 ± 23.55	0.068
TPA (°)	13.45 ± 4.88	16.81 ± 5.74	0.068
PI (°)	50.07 ± 8.15	49.84 ± 7.71	0.662
PT (°)	19.91 ± 5.18	22.02 ± 5	0.202
SS (°)	30.16 ± 9.6	27.82 ± 8.4	0.144
PJA (°)	3.1 ± 7.96	9.92 ± 8.91	0.338

*C2-FH* the distance between C2 vertical line to the femoral heads, *CL* cervical lordosis, *TK* thoracic kyphosis, *LL* lumbar lordosis, *PT* pelvic tilt, *PI* pelvic incidence, *SS* sacral slope, *SVA* sagittal vertical axis, *TPA* T1-Pelvic angle, *PJA* proximal junctional angle, *** p<0.05

**Fig. 2:**
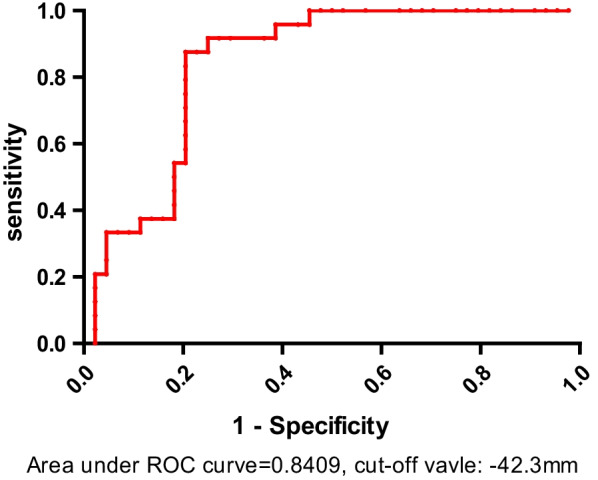
Receiver operating characteristics curve (ROC) of C2-FH

The AUC was 0.84 (95%CI 0.68–0.97), indicating the well effectiveness of ROC curve and cut-off value in predicting occurrence of PJK in ASD patients. Based on the ROC curve (Fig. 2), the optimal cut-off value of C2-FH as indicators for occurrence of PJK was − 42.3 mm. If C2-FH was smaller than − 42.3 mm, the occurrence rate of PJK was 88%. On the contrary, the rate of no PJK was 80%.

## Discussions

ASD patients are prone to be with lower back pain, functional deficit, and neurologic deficits [17]. In severe cases, surgical restoration of spinal alignment is needed to alleviate the pain and to improve the quality of life. Despite the overall satisfactory clinical outcome after surgery, unexpected mechanical complications may appear in the unfused segments and junctional region segments after surgical correction, one of which is the development of PJK. The potential risk factors of PJK have been reported by many relevant studies [18–20], however, the development of PJK is still unpredictable. The current study suggested that patients with post-operative posterior global malalignment (negative C2-FH) were more likely to develop PJK during follow-up, indicating that PJK may be a compensation for unsatisfactory global sagittal alignment restoration.

**Fig. 3:**
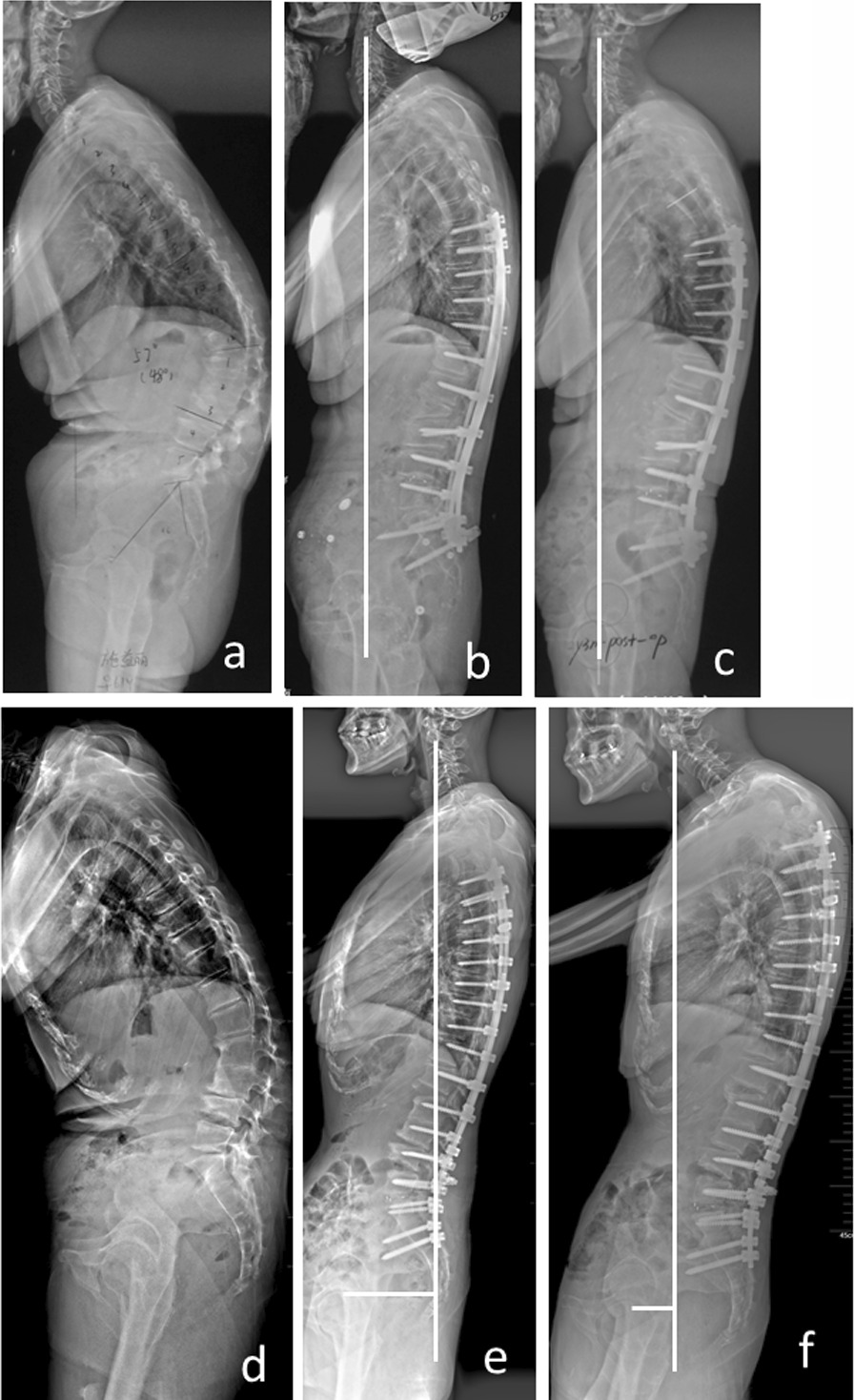
The full-length lateral films of the spine of two typical patients at immediate post-operative and at the last follow-up showed that PJK is more likely to occur for ASD patients with abnormal C2-FH at immediate post-operative (C2-FH < − 42.3 mm). The C2-FH and PJA of the first patient (A) were 0 mm and 22.2° at immediate post-operative (**b**) and 1.2 mm and 24.8° at follow-up (**c**). In another patient (B) with PJK, C2-FH and PJA were − 94.7 mm and 7.4° at immediate post-operative (**e**), while C2-FH and PJA were − 39.3 mm and 33° at follow-up (**f**). The preoperative full-length lateral films of the spine of patients A and B are shown in figure (**a**) and figure (**d**), respectively

The absolute value of post-operative C2-FH was significantly larger in PJK group compared to non-PJK group, indicating that patients with posterior global malalignment (negative C2-FH) were more likely to develop PJK (Fig. 3). The concept “posterior global malalignment” was firstly proposed by Blondel et al. [21], which was defined as post-operative SVA < 0 cm (C7 located posteriorly to the sacrum). However, they did not find the clinical significance of posterior malalignment. In our study, we use C2-FH instead of SVA to define posterior global malalignment, and the results showed that large negative C2-FH may be related to the development of PJK. C2-FH was recently proposed as a global sagittal parameter. They found that C2-FH is constant in both standing and sitting position for asymptomatic adults, the average C2-FH is − 10 mm in standing position and − 1 mm in sitting position. Thus, the stable C2-FH could be a target for sagittal alignment restoration in adults spinal deformity patients. This is the reason that we choose C2-FH, instead of SVA, to define the posterior global malalignment.

PJK was regarded as a presentation of thoracic reciprocal change after overcorrection or inducing too much lumbar lordosis. Ishihara et al. recently confirmed that overcorrection of the lumbar spine was noted as significant risk factors of PJK. Zhou et al. reported that the immediate postoperative L1-GL (gravity line) distance was associated with an increased risk of PJK after ASD surgery [22]. Their data also supported our findings since a posteriorly displaced L1 may indicate a posterior global malalignment. In addition, Wu et al. demonstrated that the more posterior the UIV was from the femoral head center, the more patients were at risk for PJK [23]. The two studies both reported the phenomenon that the more posteriorly displaced UIV (or L1), which represented overcorrection of lumbar alignment, would predispose to higher incidence of PJK, which is in accordance with our finding that posterior global malignment (posteriorly displaced C2) would predispose to the development of PJK.

Based on the “cone of economy” theory, the human body has minimal muscle work and energy consumption in a narrow upright position, suggesting that patients should develop various compensatory mechanisms to maintain a normal C2-FH. Our data also confirmed the previous report that the C2 vertebra tended to be above the center of the femoral head in asymptomatic population. At the last follow-up, the C2-FH in PJK and non-PJK group showed no significant difference (− 11.78 mm vs. − 17.96 mm), providing more solid evidence for the finding that C2-FH is a consistent parameter and could serve as a correction target. In Zhou’s study, they used the gravity line to evaluate the global alignment. The gravity line is an imaginary vertical line that passes through the whole-body center of mass. Le Huec et al. reported that the GL is located through a vertical line situated slightly to the rear of the femoral heads in sagittal plane, and Hasegawa et.al reported that during standing, the GL coincides with the vertical plumb line from the ear canal. Thus, we may assume that the normal C2-FH (C2-FH close to 0) could be regarded as a visible alternative of GL since C2 is close to the ear canal. Therefore, the normal C2-FH should be the target of sagittal compensation, and if the C2 situated far posteriorly to FH, PJK would develop as a compensatory mechanism.

Meanwhile, this study has several limitations. First and foremost, the sample size involved in this study is relatively small, so more samples are needed to verify the accuracy of predicting PJK by C2-FH and redefine the normal range of C2-FH more accurately. Second, this study was based on static lateral sagittal standing X-rays only. More recent studies have indicated the significance of muscle quality and thoracic flexibility as factors associated with development of PJK and PJF, but these could not be considered in this analysis due to the retrospective nature.

## Conclusion

Immediate postoperative negative global malalignment (C2-FH < − 42.3 mm) may predict proximal junctional kyphosis in ASD patients. The normal value of C2-FH, − 1 cm, may be the target of global sagittal compensation, and PJK is a compensatory mechanism.

## Data Availability

All data generated or analyzed during this study are included in this published article [and its supplementary information files].

## Abbreviations

ASD: Adult spinal deformity
PJK: Proximal junctional kyphosis
UIV: Upper instrumented vertebrae
TPA: T1 pelvic angle
SVA: Sagittal vertical axis
